# Genomic structure and cloning of two transcript isoforms of human Sp8

**DOI:** 10.1186/1471-2164-5-86

**Published:** 2004-11-08

**Authors:** Maria-athina Milona, Julie E Gough, Alasdair J Edgar

**Affiliations:** 1 Department of Cell Biology and Genetics, Faculty of Medicine, Erasmus Medical Center Rotterdam, PO Box 1738, 3000 DR Rotterdam, The Netherlands; 2 Manchester Materials Science Centre, University of Manchester and UMIST, Grosvenor St., Manchester, M1 7HS, United Kingdom; 3 Department of Craniofacial Development, GKT Dental Institute, King's College, Guy's Hospital, London, SE1 9RT, United Kingdom

## Abstract

**Background:**

The Specificity proteins (Sp) are a family of transcription factors that have three highly conserved zinc-fingers located towards the carboxy-terminal that bind GC-boxes and assist in the initiation of gene transcription. Human Sp1-7 genes have been characterized. Recently, the phenotype of Sp8 null mice has been described, being tailless and having severe truncation of both fore and hind limbs. They also have malformed brains with defective closure of the anterior and posterior neuropore during brain development.

**Results:**

The human Sp8 gene is a three-exon gene that maps to 7p21.3, close to the related Sp4 gene. From an osteosarcoma cell line we cloned two transcript variants that use two different first exons and have a common second exon. One clone encodes a 508-residue protein, Sp8L (isoform 1) and the other a shorter 490-residue protein, Sp8S (isoform 2). These two isoforms are conserved being found also in mice and zebrafish. Analysis of the Sp8L protein sequence reveals an amino-terminal hydrophobic Sp-motif that is disrupted in Sp8S, a buttonhead box and three C_2_H_2 _zinc-fingers. Sp8 mRNA expression was detected in a wide range of tissues at a low level, with the highest levels being found in brain. Treatment of the murine pluripotent cell line C3H10T1/2 with 100 ng/mL BMP-2 induced Sp8 mRNA after 24 hours.

**Conclusions:**

There is conservation of the two Sp8 protein isoforms between primates, rodents and fish, suggesting that the isoforms have differing roles in gene regulation. Sp8 may play a role in chondrogenic/osteoblastic differentiation in addition to its role in brain and limb development.

## Background

We are interested in understanding how Specificity proteins (Sp) govern extracellular matrix deposition during bone formation. The Sp proteins are a family of transcription factors with three zinc-fingers that bind GC-boxes and assist the further binding of the multiprotein complex TFIID promoting the initiation of gene transcription [1-4]. GC-boxes have the consensus sequence GGGCGGG. Sp1 and Sp3 are associated with chondrocytic differentiation by regulating the alpha1(II) procollagen gene (COL2A1), a chondrocytic marker. Sp1 has been shown to be an activator and Sp3 a repressor of COL2A1 transcription [5]. Despite the ubiquitous expression of Sp3, Sp3 null mice suffer from specific skeletal defects associated with a delay in osteoblast differentiation, having reduced ossification and impaired bone formation and show a decrease in the expression of osteocalcin, an osteoblast specific gene [6]. Sp7, also known as Osterix, is required for osteoblast differentiation since in Sp7 null mice bone formation does not occur [7]. Previously, we cloned two isoforms of human Sp7 that are generated from distinct promoters upstream of exon 1a and exon 1b [8]. We identified another human Sp gene that is closely related to Sp7 and named it Sp8. Recently, Sp8 null mice have been described [9,10]. Note that the mouse mBtd gene [10] is Sp8 and not a specific homologue of the *Drosophila *gene buttonhead (Btd) [11]. However, Btd is a member of the Sp family [12]. Sp8 null mice have a well-defined phenotype, having severe truncation of all limbs and no tail. Their malformed brains have defective closure of the anterior and posterior neuropores that leads to exencephaly and spina bifida. Sp8 is required for proper maintenance and maturation of the apical ectodermal ridge, a signalling centre, which forms at the limb bud apex and governs the outgrowth of the limb during development. Here we describe the sequence of two isoforms of human Sp8.

## Results

In our search for additional Sp transcription factors, we performed a TblastN search of the human genome with the sequence of Sp7 [8]. The most closely related sequences were found on genomic DNA BAC clones CTA-324D18 and CTB-86D3 that were in the draft stage (GenBank accession Nos. AC005251 and AC005060 Genome Sequencing Center, Washington University, USA). This new gene was named Sp8 since it is closely related to Sp7.

### Human Sp8 cDNAs

**Figure 1:**
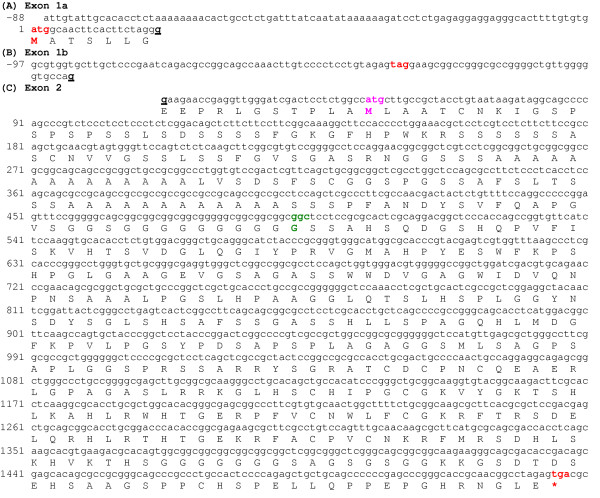
Human Sp8 cDNA sequences encompassing the ORF and translation. (A) Sequence encoding exon 1a encoding the start of Sp8L protein isoform. (B) Sequence encoding the untranslated exon 1b encoding the Sp8S protein isoform. (C) Sequence encoding the common exon 2. The atg/methionine start codon for the long protein isoform is shown in bold and coloured red and that of the short protein isoform coloured pink. The ORF stop codon, **tga**, is shown in bold and coloured red and is indicated by a red asterisk. An in-frame stop codon, **tag**, in the 5'UTR of exon 1b is shown in bold and coloured red. The exon/exon boundaries were determined by comparison with the sequence of genomic DNA clone CTA-324D18. The nucleotides underlined and shown in bold type indicate the location of the exon/exon boundaries. The loss of a GGC (glycine) codon found in an additional Sp8 clone is shown in green and bolded.

Utilising the sequences of the two BAC clones, PCR primers were designed to amplify probable cDNAs. Two different cDNA clones were obtained by RT-PCR from the human MG-63 osteosarcoma cell line and sequenced (Fig. 1). Successful amplification and sequencing required methods designed to overcome problematic GC-rich regions. These transcript variants differed at their 5' termini. Clone 1 comprises exon 1a and 2, and together they encode the long protein isoform of Sp8 (Sp8L)(Fig. 1)(GenBank accession No. AY167047). Clone 2 comprises the untranslated exon 1b and exon 2. Exon 1b contains an in-frame stop codon and no initiation methionine codon. Together they encode the short protein isoform (Sp8S)(Fig. 1)(GenBank accession No. AY167048). The first ATG codon of Sp8L is in excellent sequence consensus for an initiation methionine having a guanosine residue at positions -3 and +4, and that of Sp8S is in good sequence consensus having a guanosine at -3 [13]. The ORF is GC rich (70%). Another Sp8 cDNA clone was isolated from the osteoblasts of a patient with osteoporosis. This has a glycine-165 deletion; having lost one of the five sequential GGC codons (nucleotides 569–583) (Fig. 1).

### Human Sp8 gene

**Figure 2:**
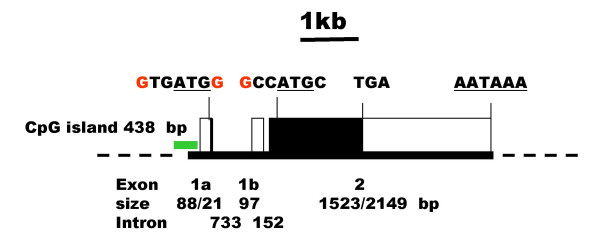
Gene structure of human Sp8. The human genomic sequences corresponding to the Sp8 cDNAs are located on clones CTA-324D18 and CTB-86D3. The location of a CpG island is shown in green. The first exon encoding the start of the Sp8L transcript is labelled 1a and that of Sp8S, 1b. The ORF is indicated by closed boxes. The sequences around the initiation methionine codons (underlined) are shown with those nucleotides corresponding to Kozak consensus sequence coloured red. The location of the TGA stop codon and a potential polyadenylation signal, AATAAA are also shown. The sizes of the exons and introns, in base pairs, are indicated.

After the completion of the human genome the location and structure of the human Sp8 gene was identified by mapping the sequences of Sp8L and Sp8S cDNA clones. The human Sp8 gene maps to 7p21.3 between the multidrug resistance protein (P-glycoprotein) and ribosomal protein L23 genes and is separated by 0.65 Mbp from the related Sp4 gene by seven genes encoding two ribosomal proteins, four putative genes of unknown function and a gene similar to argininosuccinate synthetase. The Sp8 and Sp4 genes are linked to the homeobox gene cluster HOX A, which is 5.6 Mbp away. The human Sp8 gene spans 4.6 kb and has three exons (Fig. 2). Exons 1a and 1b encode different transcript variants that are likely to be initiated from distinct promoters. The first two exons are separated by a 733 bp intron 1a, followed by a shorter, 152 bp intron 1b. All splice donor/acceptor sites contained consensus GT/AG dinucleotides. The gene possesses a CpG island upstream of exon 1a (438 bp, 63% GC). Computer analysis of the immediate human Sp8 promoter preceding exon 1a contains a number of RNA polymerase II promoter elements including a TATA-box, CCAAT-box and two GC-boxes that are conserved in rodents and fish.

### Comparison of the human Sp8 protein with those of other species

The ORF of Sp8L clone 1 encodes a 508-residue protein with a 50,500 Da molecular mass and an isoelectric point 9.02. The first ATG codon of clone 2 corresponds to the second ATG of clone 1, thereby omitting the first 18 residues of the ORF, encoding a 490-residue protein with a 48,674 Da molecular mass and an isoelectric point 9.10.

The human Sp8 protein belongs to the Sp/Krüppel like factor (KLF) family of proteins that are characterised by three Cys2-His2 zinc-fingers [14]. The zinc-fingers are located towards the COOH terminus and are involved in binding DNA in a sequence specific manner. However, the Sp proteins are distinguished from the KLF family by an amino-terminal hydrophobic domain called the Sp-motif with the consensus sequence PLALLA and a buttonhead box (BTD) with the consensus sequence CxCP(N/Y)C prior to the zinc finger domain.

**Figure 3:**
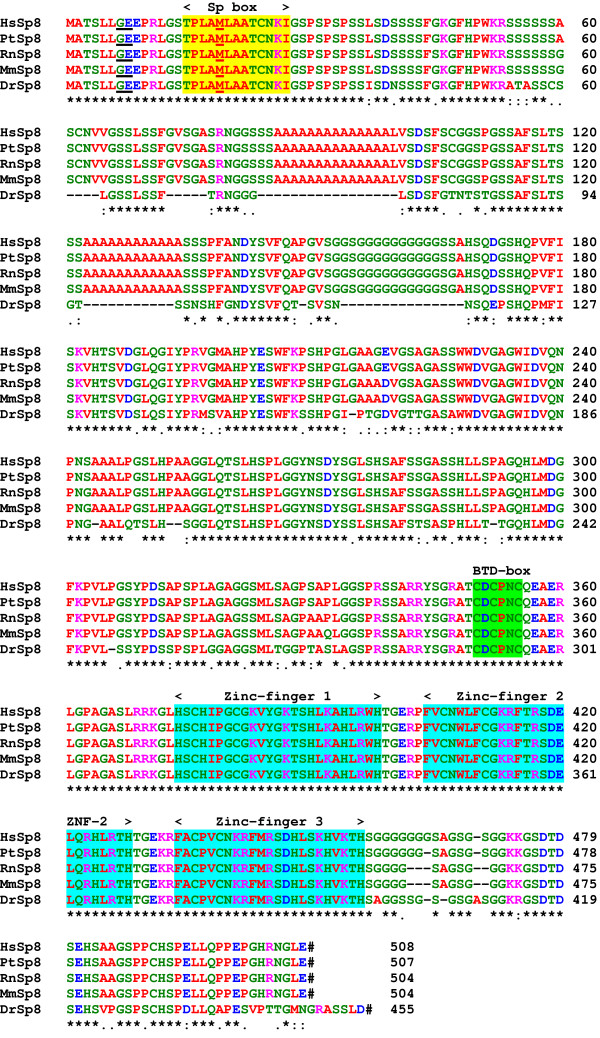
Sequence comparison of the human Sp8 protein with those from other vertebrate species. The species are man, *Homo sapiens*, Hs; mouse, *Mus musculus*, Mm; chimpanzee, *Pan troglodytes*, Pt; rat *Rattus norvegicus*, Rn and Zebrafish, *Danio rerio*, Dr. The location of the exon/exon boundaries are underlined in black on the protein sequences. The methionine residues at the start of translation of Sp8S are underlined in red. The positions of the Sp, BTD-box and three zinc-finger domains found in most Sp protein family members are indicated. The conserved residues are shown by (*), strongly conserved residues by (:) and weakly conserved residues by (.). Stop codons are indicated by a hash. Residues are colour coded: basic, DE, blue; acidic,_KR, pink; polar, CGHNQSTY, green and hydrophobic, AFILMPVW, red.

Database searches identified complete Sp8 genes and protein sequences from other vertebrate species. The chimpanzee gene is located on chromosome 7 (clone RP43-37F3); the mouse gene at 12F2 (BAC clone RP23-161L22); the rat gene at 6q33 on BAC clone CH230-1K24 and the zebrafish within BAC clone CH211-180P9. A comparison of the protein sequences shows that Sp8 is well conserved through vertebrate evolution (Fig. 3). The human and chimpanzee proteins are very similar with one conservative substitution and an additional glycine residue. The mouse Sp8 protein [9,10,15] has 97% identity and the zebrafish 79% identity to human Sp8. An amphibian Sp8 EST from *Xenopus laevis *was identified by our database search (accession No. BI313193). The mammalian Sp8 proteins differ from that of the zebrafish by the insertion of two poly-alanine and a poly-glycine region in the amino-half of the protein.

**Figure 4:**
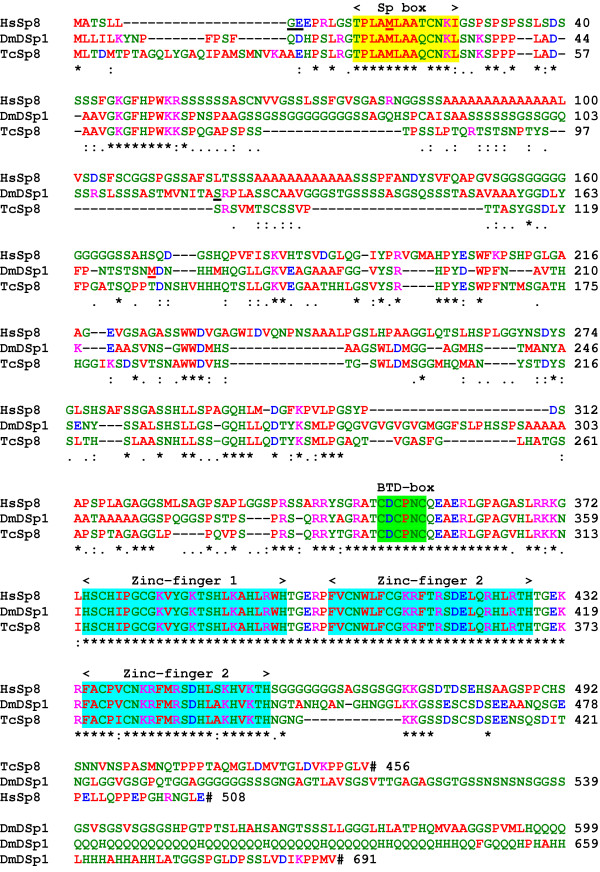
Sequence comparison of the human Sp8 protein with those from other invertebrate species; red flour beetle, *Tribolium castaneum*, TcSp8 and fruit fly, *Drosophila melanogaster *Sp8, DmD-Sp1 proteins. The locations of the exon/exon boundaries are underlined in black on the protein sequences (GE and S). The methionine residues at the start of translation for the short protein isoforms are underlined in red (M). The positions of the Sp, BTD-box and three zinc-fingers motifs are shown. Stop codons are indicated by a hash.

Database searches also showed that homologues of the vertebrate Sp8 genes are found in insects, but not in nematodes, yeasts and higher plants (Fig. 4). The fruit fly, *Drosophila melanogaster*, D-Sp1 gene is a homologue of Sp8 and not, as the name implies, Sp1 nor Sp4 as previously stated [16]. The red flour beetle, *Tribolium castaneum*, Sp8 protein [17] has 43% identity and 80% similarity to human Sp8. However, D-Sp1 differs from the vertebrate and beetle Sp8 proteins possessing a much longer, glutamine and histidine-rich carboxy-terminus. The possession of Sp, BTD and zinc-fingers domains in a protein is characteristic of Sp proteins in general; these domains in these three Sp8 proteins are very highly conserved. Outside these domains, a motif of unknown function is located towards the amino-terminal with the sequence GKGFHPWKKS and is unique to Sp8 proteins.

By comparison with other Sp proteins, the Sp8 protein can be divided up into 5 domains. The amino-terminal domain A (residues 1–53) is highly conserved in Sp8 proteins from other species. Two alpha-helices, residues 3–7 and 15–27, are predicted in this domain; whereas the rest of the protein appears to lack any secondary structure except for the zinc-finger domain. Domain A is also found in the Sp7 proteins. Domain B, residues 54–167, is a low-complexity region, having poly- serine, alanine and glycine stretches that are not present in fish Sp8 proteins. Domain C, residues 168–373, is similar to the same region of Sp7 and Sp6 that has been shown to be involved in transcriptional activation [7] and contains a basic region (residues 360–371) that probably contributes to a nuclear localization signal and a BTD-box, a highly conserved region that is present in all Sp family members. The BTD-box was first described as a 10-amino-acid region in the Drosophila zygotic gene, buttonhead [11]. Domain D, residues 374–456 contains three classical zinc finger structures (residues 374–456) of the Cys2-His2 type where the conserved cysteine residues in two short beta-sheets and the histidine residues in an alpha-helix tetrahedrally co-ordinate a zinc ion [18]. Between the fingers are five-residue linker sequences with the consensus sequence TGE+x. The negatively charged carboxy-terminal domain E, residues 457–508, contains a glycine-rich region (458–475).

### Tissue distribution of Sp8 mRNA

**Figure 5:**
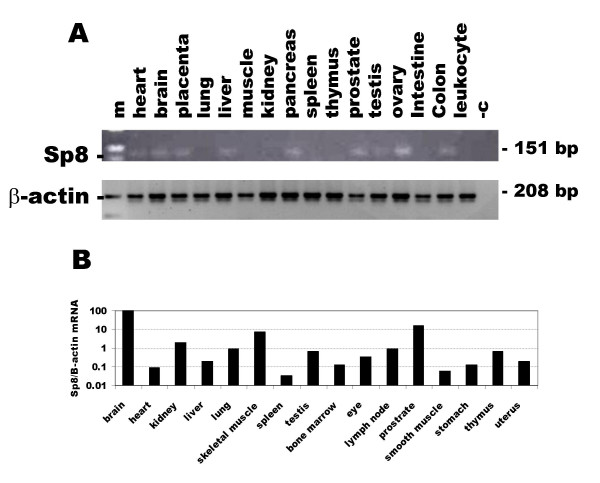
Expression of Sp8 mRNA in human and mouse adult tissues determined by RT-PCR using primers located in the 3'UTR. **A**. Ethidium bromide stained agarose gel of PCR products from human tissues. The lanes are: skeletal muscle, muscle; small intestine, intestine; peripheral blood leukocytes, leukocyte; negative control, -c and 100 bp ladder, m. The expression of beta-actin mRNA, a housekeeping gene, in the same samples is shown below. **B**. RT real time PCR of murine Sp8 mRNA expression normalised to that of beta-actin.

The expression of Sp8 in human and murine adult tissues was examined by reverse transcriptase PCR using primers located in the 3'UTR. Human Sp8 mRNA expression was detected in a wide range of tissues at a low level, being found in heart, brain, placenta, liver, pancreas, prostate, testis, ovary and colon, but was below the limit of detection in lung, skeletal muscle, kidney, spleen, thymus, small intestine and peripheral blood leukocytes (Fig. 5A). Murine Sp8 mRNA expression, relative to the expression of beta-actin mRNA, was detected in all tissues examined, with the highest levels being found in brain and prostate and the lowest in spleen (Fig. 5B).

### Expression of human Sp8 mRNA in osteoblast-like cells

**Figure 6:**
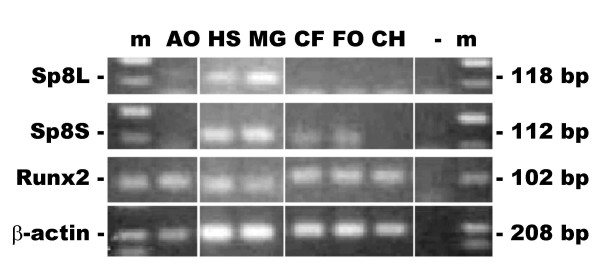
Expression of Sp8 mRNAs in human osteoblast-like cells by reverse transcriptase PCR. Amplicons were run on an ethidium bromide stained agarose gel. The amplicons were: Sp8 long protein isoform, Sp8L; Sp8 short protein isoform, Sp8S; Runx2/Cbfa1, Runx2 and the housekeeping gene beta-actin. The sizes of the amplicons are indicated. The lanes are: primary adult osteoblasts, AO; HOS osteosarcoma cell line, HS; MG63 osteosarcoma cell line, MG; primary craniofacial osteoblasts, CF; primary foetal osteoblasts, FO; primary adult chondrocytes, CH; negative control, (-) and 100 bp markers, m.

The expression of both transcript variants mRNAs encoding Sp8L and Sp8S in osteoblast-like cells was examined by reverse transcriptase PCR using primers specific for each isoform (Fig. 6). The Sp8L was most abundant in the osteosarcoma cell lines HOS and MG63 and was present in adult and craniofacial osteoblasts, but was below the limit of detection in foetal osteoblasts and chondrocytes. The Sp8S was most abundant also in the osteosarcoma cell lines HOS and MG63 and was present in craniofacial and foetal osteoblasts, but was below the limit of detection in adult osteoblasts and chondrocytes. Runx2, a transcription factor important for osteoblast differentiation, and beta-actin, a housekeeping gene, were expressed in all the osteoblast-like cells.

### BMP-2 regulation of Sp8 mRNA expression in the murine cell line in C3H10T1/2

**Figure 7:**
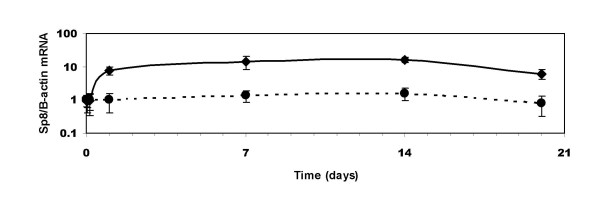
Time course of effect of 100 ng/mL BMP-2 treatment on C3H10T1/2 cells. Murine Sp8 and beta-actin mRNA expression was determined by RT real time PCR. The relative levels of expression in treated (•) and untreated cells (◆) were examined after 0, 7, 14 and 20 days treatment. Significant increases in Sp8 expression were seen at 7, 14 and 20 days (p < 0.05).

BMPs are potent secreted factors that promote osteoblast differentiation during development and bone remodelling. Since Sp8 was found in human osteosarcoma cell lines and is important in skeletal development [9,10] we examined the regulation of Sp8 by BMP-2 in the murine pluripotent embryonic cell line, C3H10T1/2 that can be induced to form a chondrogenic/osteoblastic phenotype by treatment with BMPs [19-21]. Sp8 mRNA expression, normalised to that of beta-actin, was measured by RT-real time PCR and was induced by 100 ng/mL BMP-2 after one day and remained upregulated at 20 days (Fig. 7). Data normalised to the housekeeping gene G3PDH gave similar results (data not shown) indicating that Sp8 was upregulated and that beta-actin was not down-regulated.

## Discussion

In the human genome three Sp gene pairs had been described previously; being maximally separated by 3.2 Mbp, transcribed in opposite directions, being orientated 5' to 5' manner and colocalized with a specific HOX gene clusters [8]. Sp3 and Sp5 are located on chromosome 2q31.1; Sp1 and Sp7 on 12q13.13 and Sp2-Sp6 on 17q21.3-q22. A search for a partner to Sp4 located on 7q21.3-q22 revealed a putative eighth Sp gene most similar to Sp7 suggesting that Sp8, like Sp7, may play a role also in skeletal development. Consequently, we used RT-PCR to amplify Sp8 cDNAs from a human osteosarcoma cell line and isolated two transcript variants clones that encode a full-length protein (long isoform) Sp8L and an amino-terminal truncated protein (short isoform) Sp8S, with 508 and 490 residues respectively. We found a glycine-165 deletion mutation in an additional cDNA clone isolated from a patient with osteoporosis. The length of this poly-glycine region is conserved in primates and rodents, but is absent in fish. We speculate that this Sp8 mutation, and other mutations yet to be discovered, may play a role in susceptibility to osteoporosis, and since Sp8 plays an important role in neuropore closure be a candidate gene for spina bifida [9].

The sequence of our Sp8L clone is similar to that of two other human clones, but the other two clones lack 170 and 128 bp of the low-complexity, B domain (GenBank accession Nos. BAB71297 and AAH38669). These deletions may be caused by incomplete reverse transcriptase reactions occurring during cDNA library synthesis, presumably because the high GC content (81%) generates strong secondary structure in the mRNA in this region. These deletion-carrying clones are unlikely to be generated by the presence of cryptic intron(s) since no suitable donor or acceptor splice sites are present in the genomic DNA sequence. One of the deletion-carrying clones, AAH38669, possesses a polymorphism, cac>cgc, at nucleotide 1263 that results in a His>Arg mutation at residue 448. His-448 is located in the third zinc- finger and is conserved in all Sp proteins and, by homology with Sp1, is likely to contact DNA [18]. This mutated protein would be expected to be deleterious, having reduced affinity for GC-box binding sites in promoter regions.

We found that in man Sp8 has two transcript variants utilising two different first exons. Sequence analyses of mouse and zebrafish genomic and EST data support this gene structure for Sp8 in vertebrates and not a gene structure with two untranslated 5' exons that has been suggested for murine Sp8 [10]. Interestingly, the drosophila D-Sp1 gene, a Sp8 homologue, also has two transcript variants, encoding long and short protein isoforms (accession No. AE003448) [22]. Both Sp8 and Sp7 have a similar exon structure, being three-exon genes with two transcript variants with different first exons [8]. The long protein isoforms use translated first exons that encodes seven residues and the short protein isoforms use untranslated first exons; overall, the proteins have 39.5% identity and 65.9% similarity. Both the Sp8S and Sp7S proteins lack an 18-residue amino-terminus thereby disrupting a hydrophobic region termed the Sp domain that is conserved in other Sp proteins. The conservation of the two protein isoforms through evolution suggests that they have differing roles. Although the function of this hydrophobic region is unknown, other zinc-finger transcription proteins often have a conserved protein-protein interaction domain at their amino-terminus (e.g. BTB or kelch domains). This indicates that the Sp motif may also be involved in a protein-protein interaction and that the short protein isoforms do not have this protein interaction domain.

Amino-terminal spliced variants expressed from separate promoters are a feature of other important transcription factors that regulate skeletal development. An oestrogen regulated protein in osteoblasts, KLF10 is another member of the Sp/Krüppel-like factor family that has two amino-terminal variant isoforms generated in a similar way to those of Sp8. These isoforms are named the TGFβ-inducible early gene (TIEG1) and the early growth response gene-alpha [23-25]. Runx2 has two major amino-terminal isoforms that exert different functions during the process of osteoblast differentiation; the Runx2-type-I isoform is widely expressed in osteoprogenitor cells and active osteoblasts, whereas the Runx2-type-II isoform is restricted to cells lining mineralised bones [26] and BMP-2 preferentially upregulates the Runx2-type-II isoform [27].

Sp8 does not initiate BMP-mediated signalling during apical ectodermal ridge formation, but may function downstream of the BMP receptor-1a in the signalling cascade [9,10]. The signalling events downstream of the BMP receptor that result in tissue-specific gene expression and skeletal development have been only partially elucidated. We found that Sp8 expression was induced by 100 ng/mL BMP-2 only after 24 hours in C3H10T1/2 cells. BMP-2 has been shown to induce several other transcription factors that promote differentiation such as Runx2/Cbfa1, Sp7/osterix and ZNF450 in addition to the negative regulator Id1 [7,28-30]. Induction of these genes in C3H10T1/2 cells occurs within 4 hours, preceding that of Sp8, suggesting that Sp8 is not directly regulated by BMP signalling and that it may be induced by one or more of the BMP-early-induced genes.

The Sp8 gene has a wider phylogenetic distribution than Sp7, being found in coelomates, whereas Sp7 is limited to vertebrates. In view of the close similarities between human Sp8 and Sp7 in gene structure and amino acid sequence we speculate that they evolved from an ancestral Sp8 gene during a duplication of a Sp/Hox gene cluster [31]. The Sp8 gene has retained its function in regulating appendage/limb growth [9,10,12,17] and that Sp7 has subsequently evolved a novel function, namely, regulating cartilage/bone formation [7].

## Conclusions

In humans, there are two transcript variants of Sp8 that utilise different first exons encoding long and short protein isoforms. These two isoforms are conserved being found also in the zebrafish suggesting that they have distinct functions. Sp8 is upregulated by BMP-2 in the murine pluripotent cell line C3H10T1/2 and may play a role in mesenchymal differentiation.

## Methods

### Cell culture and RNA extraction

Two cell lines derived from osteosarcomas, HOS and MG-63, were cultured at 37°C in 5% CO_2 _using Dulbecco's Modified Eagle's Medium, containing 4 mM L-glutamine, 4500 mg glucose/L, 1500mg bicarbonate/L (Invitrogen, UK) with the addition of 10% foetal bovine serum, 10 μM ascorbic acid, 100 IU/mL penicillin and 50 μg/ml streptomycin. Primary human osteoblasts were isolated from trabecular bone of femoral heads taken during total hip arthroplasty and cultured as previously described [32]. Primary human craniofacial osteoblasts were obtained from paediatric skull and cultured as previously described [33]. Human primary foetal osteoblasts were obtained and cultured as previously described [34]. Human primary articular chondrocytes were obtained from isolated femoral heads and cultured as previously described [35].

The murine cell line C3H10T1/2 was cultured in Dulbecco's modified Eagle's medium supplemented with 50 U/mL penicillin, 50 μg/mL streptomycin (Invitrogen) and 10% new born bovine serum (Sigma) at 37°C under 5% CO_2 _in a humidified incubator. Prior to BMP-2 treatment, cells were seeded at a density of approximately 40,000 cells/cm^2^, left for 24 hours, then treated with fresh media with or without 100 ng/mL human recombinant BMP-2 (Wyeth Corporation) and the media replaced every three days.

Total RNA was extracted from cells using guanidine thiocyanate and treated with DNase-I to remove any contaminating genomic DNA (SV Total RNA isolation system, Promega, UK). The concentration and purity of eluted RNA was determined spectrophotometrically and the quality of the RNA was verified by non-denaturating agarose gel electrophoresis. For Sp8 cloning, total RNA was reverse transcribed with an oligo-dT primer using ThermoScript, an AMV RNase H- reverse transcriptase at an elevated temperature of 60°C (Invitrogen, UK).

### Molecular cloning of human Sp8

The human Sp7 protein sequence [8] was used to search the human genome sequence and the EST database to identify closely related genes. PCR primers designed from the sequence of genomic DNA clones CTA-324D18, CTB-86D3 and EST sequence from Image clone 2721342 (accession No. AW207154) were used to clone two transcript variants spliced isoforms of the human Sp8 cDNA by PCR from MG-63 osteosarcoma cell line cDNA. The PCR primers were: Sp8L forward, ATTGTATTGCACACCTCTAAAAAAAACA; Sp8S forward, GCGTGGTGCTTGCTCCC and common reverse, GCGTCACTCTAGGCCGTTG (Helena Biosciences, France). The cDNAs were amplified by PCR with an annealing temperature of 60°C using the Advantage-GC Advantage kit (Clontech, UK) with the addition of 0.5 M GC-Melt since the DNA sequence of Sp8 is GC-rich. PCR products were excised from agarose gels stained with ethidium bromide and eluted from the agarose using a DNA extraction kit (Qiagen, UK). The PCR products were cloned into the T-A vector pCR4-TOPO (Invitrogen, The Netherlands). Transformed colonies were picked and vectors containing inserts were extracted using the Wizard Plus SV minipreps DNA purification system (Promega, UK) and sequenced in both directions using ThermoFidelase 2 (Fidelity Systems Inc., USA).

### Tissue and cellular distribution of human Sp8 mRNA by reverse transcriptase PCR

Human cDNA was analysed for the relative expression of the Sp8, Runx2/Cbfa1 and beta-actin mRNA by PCR. Sixteen adult tissue cDNAs (BD Clontech, UK) were generated from polyA^+ ^selected RNA and reverse transcribed using an oligo-dT primer. Total RNA from cell type cDNAs were reversed transcribed using random hexamer primers using an AMV RNase H- reverse transcriptase (ThermoScript, Invitrogen, UK) at 60°C. Approximately 4.0 ng of cDNA from each tissue, and cDNA derived from 50 ng of total RNA from each cell type were amplified by PCR using Taq Gold polymerase. Tissue master mixes were divided into gene specific mixes with the addition of PCR primers to a final concentration of 200 μM. The primers were: Sp8L forward primer, GCAACTTCACTTCTAGGG**GAAGA**(exon 1a/**2**); Sp8S forward primer, TGGGGGTGCCAG**GAAGAAC**(exon 1b/**2**) and a common reverse primer, AGCTGTCCGAGAGGGAGGA (exon 2), producing a 118 and 112 bp amplicons respectively; Sp8 3'UTR, TTAGTCCGGCCGTCAATTGT and TGGTATTTAAACTACAGCCTCGTCTGA, producing a 151 bp amplicon; Runx2, AGAAGAGCCAGGCAG**GTGCT**(exon 6/7) and TTCGTGGGTTGGAGAAGCG (exon 7), producing a 102 bp amplicon, as measure of the sum of all Runx2 isoforms, and beta-actin, GGCCACGGCTGCTTC and GTTGGCGTACAGGTCTTTGC, producing a 208 bp amplicon. The amplification conditions were; a 10 min hot start to activate the polymerase followed by 50 cycles of 95°C for 15 sec and 60°C for 1 min. Amplification specificity was confirmed by direct sequencing of the amplicons.

### Expression of murine Sp8 mRNA by real time PCR

Utilising a CAS-1200 robotic precision liquid handling system, PCR was carried out on a Rotor Gene 3000 (Corbett Research, Australia) using a SYBR Green I double-stranded DNA binding dye assay. Copy DNA derived from 200 ng of total RNA from each sample was amplified by PCR using Taq Gold polymerase using PCR primers to a final concentration of 50 nM. The primers were: mouse Sp8 3'UTR, CCATTCAGCTCTGGCTAGGTCTT and GATTCCCGTTCGCAGAACTC producing a 67 bp amplicon. Beta-actin mRNA was used as a control gene as previously described [36]. The amplification conditions were; a 10 min hot start to activate the polymerase followed by 40 cycles of 95°C for 15 sec and 60°C for 1 min. The number of cycles required for the fluorescence to become significantly higher than background fluorescence (termed cycle threshold [C_t_]) was used as a measure of abundance. A comparative C_t _method was used to determine gene expression. Expression levels in each cDNA sample were normalised to the expression levels of the control gene (ΔC_t_). The ratios of Sp8 mRNA/control gene RNA from each cDNA were standardised to that of the untreated cells on day 0 that was taken as 100% (ΔΔC_t_). The formula E^-ΔΔCt ^was used to calculate relative expression levels where E is the efficiency of the PCR per cycle. Statistically significant changes in gene expression were determined using the t-test on data from three replicate experiments. The amplification specificity was confirmed by melting curve analysis and agarose gel electrophoresis.

## Authors' contributions

AJE conceived of the study, and participated in its design and coordination. MAM, JEG and AJE carried out the cell culture work, MAM and AJE carried out the expression studies and MAM and AJE carried out the gene cloning. All authors participated in writing the manuscript, read, and approved the final manuscript.
